# Supplementary material for: Are carbon emissions trading and green financial instruments synergistic? -Comprehensive quantitative research based on content analysis
Source: PLoS One. 2024 Mar 7;19(3):e0298601. doi: 10.1371/journal.pone.0298601 (PMC10919601; doi:10.1371/journal.pone.0298601)
Supplement: S1 Data — (ZIP) [file pone.0298601.s001.zip › Data/地级市Z_F工作报告.zip!/╕ú╜¿/╚2├≈╩╨/└┤╫╘╣1⁄2╓┌║┼í╛┬φ┐╦╩2╛▌═°í┐_2019.docx]

各位代表：

　　现在，我代表三明市人民政府向大会作政府工作报告，请予审议，并请市政协各位委员和其他列席人员提出意见。

　　一、2018年工作回顾

　　过去一年，全市各级政府各部门深入学习贯彻习近平新时代中国特色社会主义思想和党的十九大精神，全面贯彻落实党中央国务院、省委省政府和市委决策部署，迎难而上，扎实工作，经济社会发展总体保持稳中有进态势。预计全市生产总值2332亿元，增长7.4%；规模以上工业增加值910亿元（同口径），增长8.8%；地方一般公共预算收入107.64亿元，增长6.83%；固定资产投资增长12%；外贸出口161亿元，增长9.6%；社会消费品零售总额592.11亿元，增长11%；城镇居民人均可支配收入34874元，增长8.1%；农村居民人均可支配收入16581元，增长9%；居民消费价格总水平上涨1.6%。一些重点领域和项目取得新突破：

　　——重大项目支撑有力。南龙铁路建成通车，莆炎高速全线开工，浦梅铁路、兴泉铁路、山水林田湖草生态保护修复工程试点等重大基础设施项目加快推进。实施“一十百千万”工程，谋划实施10亿元以上重大产业项目105个、总投资1378.5亿元，其中，总投资31.1亿元的三化高纯超净电子级氟化氢、总投资24亿元的三明化机高端智能注塑机等项目开工建设，总投资18亿元的三钢闽光物联云商、总投资10.5亿元的厦钨新能源三元正极材料、总投资7.2亿元的明一奶粉、总投资5亿元的海斯福（三期）锂电池电解液等项目建成投产。

　　——重点改革先行先试。在全国率先推行全民健康“四级共保”工程、医保基金打包支付和C-DRG收付费改革，三明市、将乐县公立医院综合改革成效受到国务院督查激励，我市“建设紧密型医联体，向全民健康再出发”医改典型经验受到国务院第五次大督查通报表扬；持续创新普惠性金融产品，“福林贷”“快农贷”模式在全国推广；在全省率先推行以排污权抵押贷款、设立碳汇基金、推行环境污染责任保险为主要内容的绿色金融改革，成功举办绿色金融论坛。

　　——生态质量全省前列。市区空气质量达标率99.2%，全省第一；将乐、泰宁、建宁、明溪、清流、宁化、大田等7个县空气质量进入全省58个县级城市前十名，数量全省第一；全市110个小流域断面Ⅰ-Ⅲ类水质比例96.4%，全省第一；18个国（省）控断面水质100%达到或优于Ⅲ类水质，全省第一；全市18个县级以上饮用水源地100%达到或优于Ⅲ类水质，全省第一；在全省率先消除Ⅳ类及以下水体。“尤溪联合梯田”入选联合国粮农组织全球重要农业文化遗产，将乐跻身第二批国家生态文明建设示范县，永安荣获“2018中国最具生态竞争力城市”称号。

　　——营商环境持续改善。组织开展“六最”营商环境对标活动，全面推进“证照分离”“多证合一”“多审合一”改革工作，审批和服务时限控制在法定时限的20.2%以内，为全省最短；市级不动产登记从原来的15个工作日压缩至5个工作日以内；全市企业开办时间压缩至4个工作日以内，比省政府的要求少4天。

　　——民生保障更多实惠。28项省市为民办实事项目全面完成，提前两年基本消除乡镇和农村公共旱厕；在全省率先推行精准扶贫医疗补助政策，建档立卡贫困人口在本市范围内（外地医保目录内）看病每人每年自付300元封顶；全面实现低保、特困供养标准城乡一体化；“总校制”改革县级试点有序推进，高考再创佳绩，普惠性学前教育资源覆盖率居全省前列。

　　过去一年的主要工作和成效是：

　　一年来，我们着力落实赶超，经济发展稳中提质。研究出台《实施“一十百千万”工程推动高质量发展奋力实现赶超的意见》，制定实施百亿龙头企业、千亿产业集群培育方案和培育壮大百亿龙头企业八条扶持措施，全力推动高质量发展落实赶超。深入推进供给侧结构性改革，全市兼并重组企业4家，压减煤炭产能135万吨，帮助企业降低各类成本13亿元。加强科技研发平台建设，机械科学研究总院海西分院实施新一轮“三方共建”，成立中国医药工业研究总院三明分院、三明新能源产业技术研究院，我市12项成果获省科技进步奖、5项专利获省专利奖。实施新一轮技改提升、战略性新兴产业倍增、现代服务业创新发展、现代农业提升发展四个行动计划，冶金及压延、汽车及机械装备、林产加工、纺织四大传统产业实现产值2488.56亿元、比增10.2%，稀土、氟新材料、石墨（烯）、生物医药等战略性新兴产业实现产值780亿元、比增20%。旅游、文化、体育、健康等热点消费加快发展，新增限上商贸企业和规上服务业企业90家，电子商务网上零售额增长24.7%；粮食安全省长责任制工作走在全省前列，新增“三品一标”农产品33个，高优粮食、绿色林业、精致园艺、生态养殖、现代烟草等5大特色产业实现产值1400亿元，增长8%。

　　一年来，我们着力攻坚克难，瓶颈难题有效破解。全力打好项目攻坚战，深入开展“五比五晒”项目竞赛，组织开展“五个一批”项目攻坚冲刺100天行动，新增省级“五个一批”项目3660个、总投资4523.1亿元，新增项目数居全省第一位，其中新增开工项目976个、居全省第二位，新增增资项目180个、居全省第一位；强化“一把手”招商、市县联合招商，建立市政府招商顾问制度，全年共签约项目1077个、总投资1071.6亿元。全力打好“三大攻坚战”，持续深入开展金融案件审判执行、打击恶意逃废债、公职人员不良清欠三大专项攻坚行动，全年化解不良贷款40亿元，不良率降至1.91%，地方政府债务控制在省核定的限额范围内；加快国家扶贫改革试验区建设，组织开展扶贫资金专项检查、造福工程易地扶贫搬迁专项检查、扶贫领域作风专项治理、贫困户大走访四项重点工作，建档立卡贫困人口全部脱贫，预计2个重点县、14个重点乡、220个建档立卡贫困村、122个贫困空壳村可实现脱帽；抓好“三合一”环保督察整改，深入推进以“工业污染、畜禽养殖污染、城乡生活污水垃圾、涉砂行为、小水电生态”为重点的水环境综合治理，组织开展“淘汰落后产能、矿山整治、农村环境综合治理”三个专项行动，深化河湖长制，在省政府落实最严格水资源管理制度考核中连续四年被评为“优秀”。全力打好市区“城市双修”、“和谐征迁”攻坚战，三明学院附属小学扩容、沙溪西岸自行车道、三钢省级观光工厂、龙岗夜市等项目建成，城市渣土车“跑冒滴漏”、“二手车”占道经营等一批难点问题有效治理，完成土地征收3017亩、房屋征收651户、企业征收12家，城市发展环境得到进一步改善。

　　一年来，我们着力协调发展，城乡建设有力推进。主动融入闽西南协同发展区建设，谋划实施了13个、总投资527.36亿元的重大协作项目，与厦门对接了52个、总投资108.12亿元的厦企项目。推动市区经济加快发展，抓好九个专业特色工业园区建设，154个市区经济重点项目完成投资107.12亿元，翔丰华二期、三农含氟新材料及配套中间体、厦工传动设备等一批项目开工建设或部分投产。持续壮大县域经济，永安保持全省县域经济实力“十强”县、建宁保持县域经济发展“十佳”县，5个省级扶贫开发工作重点县加快发展，尤溪洋中镇省级“小城市”改革试点和5个省级特色小镇建设进展顺利。编制《三明市实施乡村振兴战略规划（2018—2022年）》，开展125个村庄环境整治，建设12个美丽乡村示范村、26个省级乡村旅游村，永安通过全国“四好农村路”示范县命名复核，宁化水土保持科技示范园列入全国水土保持科普教育基地。实施城乡基础设施补短板项目969个，全市新开工及续建国省干线道路100公里，改扩建农村道路343公里，新建改建污水管网150公里，新增及改造城乡配电网1203公里，新增城乡公共停车泊位7309个，新建改建公厕615座。

　　一年来，我们着力改革开放，动力活力持续释放。巩固提升“三医联动”改革成果，“总医院”运行机制逐步完善。持续深化林业改革和金融改革，创新推广“福田贷”等金融产品，全年新增园区企业资产按揭贷款26.52亿元，新增林权抵押贷款5.6亿元。有序推进农村土地“三权分置”改革，基本完成承包地确权登记颁证任务，土地承包经营权累计流转面积81.5万亩，全面推进农村集体产权制度改革，农村集体清产核资工作基本完成。全面完成全国小微企业创业创新基地城市示范任务，永安市、建宁县入选全国农村创业创新典型县。有序推进市、县机构改革，完成承担行政职能的事业单位改革试点工作。明港澳台侨及对外交流合作持续深化，成功举办第十四届林博会，国际友城交流取得新成效，与“一带一路”国家和地区交流合作更加紧密。

　　一年来，我们着力补齐短板，社会事业加快发展。去年全市民生支出234.77亿元，占一般公共预算支出的79.18%。社会保障体系不断完善，全市新增城镇就业1.84万人，保障性安居工程新开工725套，基本建成1496套。实施基础教育补短板项目118个，新增中小学学位5600个、幼儿园学位7020个。深化与高水平医院协作，新增省级临床重点建设学科4个，启动实施三明中医振兴发展工程。三明市被列入第三批全国居家和社区养老服务改革试点市，宁化、泰宁、建宁被列为农村养老服务改革试点县。全面开展“周周有戏看”“月月有比赛”“半台戏”等群众性文体活动，我市运动员在亚运会、亚残运会分别取得1金1银、2金2银1铜的好成绩，成功承办福建省第九届少数民族传统体育运动会、举办市第十届老年人体育健身运动会。深入开展扫黑除恶专项斗争和五个专项治理，群众安全感率、执法工作满意率均居全省前列。积极化解信访积案，扎实推进“七五”普法。强化安全生产领域改革发展，防灾减灾救灾能力进一步提高。深入实施治理“餐桌污染”，建设“食品放心工程”，“一品一码”追溯体系有效推进。巩固提升全国文明城市创建成果，三明市被列入全国“志愿之城”试点城市，通过“国家卫生城市”复审。村（居）委会换届工作全面完成。第四次全国经济普查全面启动。老龄、工会、青少年、妇儿、残疾人工作持续推进，科普、民族宗教、档案、地方志、水文、气象、地震、库区移民、对口援疆工作扎实开展。双拥共建和人民防空、国防后备力量建设深入推进。

　　一年来，我们着力优化服务，自身建设不断加强。全面兴起习近平新时代中国特色社会主义思想“大学习”热潮。强化全面从严治党，加强廉洁政府建设，认真贯彻落实中央八项规定及实施细则精神和省、市实施办法，进一步加强审计监督，严肃查处顶风违纪行为和“四风”隐形变异问题。践行“马上就办、真抓实干”，深入推进机关效能建设，持续深化“千名干部服务千家企业”活动，在全省率先成立市非公企业服务中心。加快推进法治政府建设，自觉接受人大法律监督和工作监督、政协民主监督，高度重视、认真办理人大代表建议和政协提案，支持民主党派、工商联和无党派人士参政议政，提请市人大常委会审议《三明市城市园林绿化管理条例》，出台《三明市建筑垃圾管理办法》。深化“放管服”改革，共取消、下放、调整市级权责事项213项，梳理“最多跑一趟”事项363项、“一趟不用跑”事项291项，市级38个单位的745项审批和服务事项入驻网上办事大厅。

　　各位代表，过去一年成绩来之不易，这是省委省政府和市委坚强领导的结果，是全市人民团结奋斗的结果，是社会各界大力支持的结果。在此，我代表市人民政府，向为三明建设发展付出辛勤劳动的全市人民，向给予政府工作有力支持与有效监督的人大代表和政协委员、各民主党派、工商联、各人民团体、无党派人士、离退休老同志及社会各界人士，向积极发挥作用的中央和省属驻明单位、驻明部队、武警官兵、政法干警，向关心支持三明发展的港澳台同胞、海外侨胞、国际友人，表示衷心的感谢！

　　我们也清醒地认识到，全市经济社会发展还存在不少困难和问题，主要是：经济运行稳中有变，下行压力加大；产业转型升级不够快，高新技术产业和新兴产业规模偏小，现代服务业发展偏慢；部分中小微企业经营困难，融资成本高、创新能力弱等问题较突出；大项目、税源型项目还不多，项目服务保障有待加强；区域发展还不够协调，新型城镇化质量有待提高，中心城市辐射带动能力不强，教育、卫生、养老等民生短板仍较突出，营商环境还需进一步提升；部分干部干事创业激情和担当精神有待增强，有些领域形式主义、官僚主义问题仍不同程度存在，等等。这些问题是前进中的问题，我们一定高度重视，有针对性地加以解决。

　　二、2019年工作安排

　　今年是新中国成立70周年，是决胜全面建成小康社会、推进高质量发展落实赶超的关键一年。我们要紧紧把握我国发展重要战略机遇期，咬定目标不放松，坚定信心不动摇，应对一切困难，拿出百倍努力，创造更大业绩，为全面建成小康社会打下决定性基础，以优异成绩庆祝新中国成立70周年。

　　今年政府工作总体要求是：以习近平新时代中国特色社会主义思想为指导，全面贯彻党的十九大和十九届二中、三中全会精神，统筹推进“五位一体”总体布局，协调推进“四个全面”战略布局，坚持稳中求进工作总基调，坚持新发展理念，坚持高质量发展落实赶超，坚持以供给侧结构性改革为主线，坚持深化市场化改革、扩大高水平开放，继续打好“三大攻坚战”，统筹推进稳增长、促改革、调结构、惠民生、防风险工作，进一步稳就业、稳金融、稳外贸、稳外资、稳投资、稳预期，提振市场信心，增强人民群众获得感、幸福感、安全感，努力把三明建设成为绿色发展先行区、产业转型集聚地、深化改革排头兵、区域协同桥头堡、宜居宜业生态城。今年发展主要预期目标是：地区生产总值增长8%左右；地方一般公共预算收入增长3%；固定资产投资增长12%；外贸出口增长3%；实际利用外商直接投资增长3%；社会消费品零售总额增长11.5%；居民消费价格涨幅3%左右；城镇登记失业率控制在4.2%以内；城镇居民人均可支配收入增长8.5%，农村居民人均可支配收入增长9%；单位生产总值二氧化碳排放降低目标控制在省下达范围内；完成能源消耗总量及强度“双控”目标和主要污染物减排年度任务。围绕上述目标，重点抓好六个方面工作：

　　（一）大力推进高质量发展落实赶超。坚决贯彻落实省、市既定部署，对照时间表、落实施工图，加大力度、加快进度，千方百计推动高质量发展落实赶超取得更大成效。

　　深入实施“一十百千万”工程。进一步完善23个产业园区基础设施，加快推进105个投资10亿元以上重大产业项目建设，全年完成投资206亿元以上，三明信息经济产业园、三化年产5万吨电子级氟化氢等一批项目竣工投产。加快实施三明市百亿龙头企业培育实施方案，落实培育壮大百亿龙头企业八条扶持措施，推动一批企业尽快成为“金娃娃”；加快推进五大千亿产业集群培育计划，力争林产加工、冶金及压延、汽车及机械装备、纺织、新材料五大产业产值分别实现930亿元、900亿元、875亿元、800亿元、600亿元以上。巩固提升全国小微企业创业创新基地城市示范建设成果，着力扶持万家小微企业创业创新，新增小微企业6000家以上。

　　加快创新型城市建设。加强科技创新投入，力争全社会研究与试验发展经费投入增长22%。大力培育“双高”企业、科技小巨人企业、“专精特新”企业，力争新增省级高新技术企业30家、国家级高新技术企业20家。加强科技研发平台建设，继续抓好机械科学研究总院海西分院、氟化工产业技术研究院、新能源产业技术研究院、石墨（烯）产业技术研究院、三明农科院、中国医药工业研究总院三明分院等六大科技研发平台建设，不断提升科技研发水平。弘扬劳模精神、工匠精神，大力引进和培养科技创新型人才，深入实施“海纳百川”高端人才聚集计划，用好院士专家工作站，让各类优秀人才多起来、用得好、留得住。

　　深化“五个一批”项目建设。发展千条万条，没有项目就是白条。开展推进高质量发展落实赶超竞赛活动，狠抓项目谋划、开工、投产关键环节，完善重大项目挂牌服务、“一月一协调、一季一督查、一季一通报”机制，持续开展“和谐征迁”攻坚行动，及时协调解决用地、用林、融资和环境容量等问题，力争全市新增“五个一批”亿元以上项目1000个以上。突出抓好重大基础设施项目建设，加快浦梅铁路、兴泉铁路和莆炎高速公路三明段建设，莆炎高速永泰梧桐至尤溪中仙三明段建成通车，力争开工武夷新区至沙县高速公路三明段，深化3条高速公路联络线和昌厦（福州）高铁、鹰潭经资溪至建宁铁路等项目前期工作，实施安全生态水系、防洪工程、流域综合整治工程等水利项目，抓好海西天然气管网三明段、农村电网改造升级等能源项目，推进三明核电、抽水蓄能发电等项目前期工作。

　　（二）大力推进实体经济发展。把振兴实体经济作为供给侧结构性改革的主要任务，着力推动制造业高质量发展，着力破除体制性障碍，着力优化营商环境，坚定不移把实体经济抓上去。

　　深化供给侧结构性改革。巩固“三去一降一补”成果，增强微观主体活力，提升产业链水平，畅通经济良性循环。落实新一轮技改提升行动计划，实施市级重点技改项目300个、完成投资200亿元以上，促进冶金及压延、汽车及机械装备、林产加工、纺织四个传统产业向高端化、智能化、绿色化、集群化发展。落实战略性新兴产业倍增计划，坚定不移推动资源往产业走，加快实施稀土分离、石墨负极材料、高端氟精细化学品等产业链重点项目，推动稀土、石墨(烯)、氟新材料、生物医药四个战略性新兴产业加快发展。落实现代服务业创新发展行动计划，致力服务消费与实物消费同步发展，加快推动教育、育幼、养老、医疗、文化、旅游等服务消费提质扩容，引导食品、汽车、电子产品等实物消费提档升级，推进国家全域旅游示范区建设，抓好制造业与现代服务业深度融合、现代服务业集聚区建设、平台经济发展等工作，加快三明陆地港冷链物流区、中通快递闽赣区域总部等项目建设，促进服务业质量提高、比重提升。落实现代农业提升发展计划，严格落实粮食安全省长责任制，确保“饭碗端在自己手上”，持续推进高优粮食、绿色林业、精致园艺、生态养殖、现代烟草等5大特色产业、20条产业链发展，大力实施“五百工程”，进一步提升现代农业发展水平。

　　优化实体经济发展环境。毫不动摇鼓励支持引导民营经济发展，真正把民营企业和民营企业家当自己人。进一步清理、精简涉及民间投资管理的行政审批事项和涉企收费，规范中间环节、中介组织行为，减轻民营企业负担。坚决打破各种各样的“卷帘门”“玻璃门”“旋转门”，推进产业政策由差异化、选择性向普惠化、功能性转变，为民营企业打造公平竞争环境。落实和完善促进民营企业健康发展政策措施，有效破解企业融资、市场、技术等难题，让民营企业从政策措施中增强获得感。构建亲清新型政商关系，深入开展“千名干部服务千家企业”活动，营造“懂民企、爱民企、帮民企”良好氛围。依法保护企业家人身安全、财产安全，保障企业合法经营，让企业家卸下思想包袱、轻装前进。深化“放管服”改革，全面推行审批服务“马上办、网上办、就近办、一次办”。深入开展“六最”营商环境对标活动，凡是不与现行法律法规相抵触、可以取消和下放的行政许可事项一律取消和下放；凡是全省其他设区市未列入审批或许可的事项，一律不列入审批；凡是其他同类地区营商环境方面可以做到的，原则上一律做到，为实体经济发展创造更加良好服务环境。

　　强化招商引资实效。完善招商机制，充分发挥市七个项目招商工作组和市政府招商顾问作用，突出“一把手”招商和市县联合招商，深化明商“回归工程”，用好“6·18”“9·8”等招商平台，力争全年签约项目投资总额达1400亿元。突出招商重点，持续加强与世界500强和央企、省企、民企、外企对接，实行“一对一”全程跟踪服务，力争中国通用集团竹天丝、中核集团产业园等一批合作项目加快落地。创新招商形式，推动以商招商、产业链招商、专业招商，探索产业基金招商、第三方招商，促成更多招商成果落地。

　　（三）大力推进三大攻坚战。始终保持攻坚克难的锐气，聚焦突出问题，打好重点战役，专组负责、挂图作战、紧盯不放，保质保量完成各项攻坚任务。

　　坚决打好防范化解重大风险攻坚战。完善地方金融监管工作机制，开展互联网金融风险专项整治，规范地方金融和类金融机构发展，守住不发生系统性金融风险的底线。严厉打击非法集资等违法违规行为，切实帮助群众守住血汗钱、救命钱、养老钱。强化金融业服务实体经济能力，支持优质企业挂牌上市、园区资产按揭贷款等金融创新，加大重点项目、民生工程、中小微企业等信贷投放力度，新增贷款130亿元以上。规范政府举债行为，积极争取地方政府专项债券支持，严格控制地方政府债务规模。健全完善房地产市场平稳健康发展长效机制，坚持“房子是用来住的、不是用来炒的”定位，落实城市主体责任，因城施策、精准调控，切实稳地价、稳房价、稳预期。

　　坚决打好精准脱贫攻坚战。扎实推进脱贫攻坚战三年行动，打好产业扶贫稳定增收、就业扶贫务工增收、教育扶贫、健康扶贫、住房安全保障等五大战役，全面落实贫困人口“两不愁三保障”，确保今年29个建档立卡贫困村、31个贫困空壳村、2个市级扶贫开发工作重点乡、2个省级扶贫开发工作重点县实现摘帽退出。深化国家扶贫改革试验区建设，提升“348”精准扶贫机制，推广市区“城市困难家庭精准帮扶试点”经验，进一步探索精准脱贫长效机制，为全省全国创造可复制、可推广的三明经验。坚持脱贫不脱责任、脱贫不脱政策、脱贫不脱帮扶、脱贫不脱监管，“扶上马送一程”，巩固提升脱贫成果。

　　坚决打好污染防治攻坚战。突出大气、水、土壤污染防治，深化“工业污染、畜禽养殖污染、城乡生活污水垃圾、涉砂行为、小水电生态”五项攻坚和“淘汰落后产能、矿山整治、农村环境综合治理”三项行动，加快三钢节能减排等重点项目建设，加快金溪、沙溪、尤溪三大主要流域水环境治理与生态修复，加快土壤环境保护省级示范区建设，确保我市各流域断面均值稳定达到或优于Ⅲ类标准，空气质量达标天数比例继续走在全省前列，土壤安全利用率有明显提升。大力推动绿色经济低碳循环发展，提高绿色产业技术装备水平，推进闽江流域山水林田湖草生态保护修复试点建设，完成国土绿化和森林经营195万亩、水土流失治理面积38万亩。

　　（四）大力推进区域协调发展。坚定不移走区域协调发展战略，加大“统”的力度，做好“融”的文章，加快“同”的步伐，把城乡一体化发展推向更高水平。

　　主动融入闽西南协同发展区建设。认真落实《闽西南协同发展区发展规划》和《三明市主动融入闽西南协同发展区建设行动计划（2018—2020年）》，抓项目协作，加快推进13个重大协作项目和52个厦企项目，积极融入闽西南航空经济圈，推动“陆地港”与沿海港口功能全面对接；抓平台建设，支持5个省级扶贫开发工作重点县和大田、永安等地与沿海共建产业园，促进产业紧密“联姻”；抓机制创新，对接闽西南协同招商机制，参与组建闽西南城市协作开发集团和“福建两翼发展投资基金”，推动完善跨区域生态补偿机制，不断增强协同发展实效。

　　进一步提升市区发展水平。着力推动市区产业强起来，深入实施市区经济重点项目建设行动计划，抓好九个专业特色工业园区建设，加快推进三钢打造千亿集团、三明化机高端智能注塑机、翔丰华三期等重点工业项目建设，加快推进万寿岩遗址公园、三明郊野国家地质公园等重点服务业项目建设，加快推进一批城郊型农业项目建设，进一步提升市区产业发展水平。着力推动市区环境美起来，深入推进“城市双修”，重点实施市区老旧小区微改造、沙溪河生态修复、“两沿一环”森林景观带改造提升、市区绿化提升、贵溪洋生态湿地公园、市区公共停车设施等示范项目，推进智慧城市建设，开展第二轮“两违”综合治理行动，加快打造现代山水名城。着力推动市区社会事业好起来，进一步提升市区中小学和幼儿园教育水平，加快打造市区优质教育高地，加强市第一医院和中西医结合医院医疗服务能力建设，打响“周周有戏看”文化品牌，不断提升市区公共服务供给水平。着力推动市区空间大起来，持续推进市区与沙县、永安同城化一体化，抓好城市快速通道二期、534国道槐林至荆东段、205国道台江连接线等项目建设，加快北部新城、南部新城和生态新城“一带六区”“五大板块”开发建设，统筹抓好洋溪、陈大、莘口、岩前等市区周边小城镇开发建设，进一步拉开城市框架。

　　加快发展县域特色经济。落实加快县域经济发展政策措施，支持各地从区位条件、资源禀赋和产业基础出发，大力发展特色产业，办好重点工业园区，走产城融合路子。支持永安、沙县争创全省县域经济实力“十强”县，支持将乐、尤溪、大田争创全省县域经济发展“十佳”县，支持明溪、清流、宁化、建宁、泰宁加快脱贫和稳定脱贫。推进“大城关”建设，强化县城规划引领，加快新区开发和旧城改造步伐，进一步完善配套功能，抓好已在城镇就业的农业转移人口落户工作，提升城镇化发展水平。

　　推进乡村振兴战略实施。突出产业振兴，坚持走特色农业“接二连三”路子，加快农产品质量安全市建设，持续打响“尚书品”“食尚三明”等公共品牌和宁化河龙贡米、建宁莲子等一批特色农业品牌，推进农产品精深加工和流通，发展农业旅游、“互联网+现代农业”等多种新型业态，全年新增“三品一标”农产品20个以上。注重人才振兴，落实好科技特派员制度，鼓励农民工返乡创业和高校毕业生、企业主、农业科技人员等各类人才下乡创业，实施新型职业农民培育工程，着力培养一支有文化、懂技术、会经营的新型职业农民队伍。实施文化振兴，加强农村思想道德建设，强化历史文化资源和传统村落保护与传承，推进新时代文明实践中心建设，大力开展移风易俗活动，提升乡村文明水平。狠抓生态振兴，深入实施“千村整治、百村示范”工程，全面开展农村人居环境整治三年行动，着力抓好“一革命五行动”，加快向莆铁路、南龙铁路沿线综合整治，所有乡镇生活污水处理设施基本建成并投入使用，改扩建农村公路200公里以上。强化组织振兴，大力扶持发展农民合作社、家庭农场等新型经营主体，积极培育服务性、公益性、互助性农村社会组织，推进“一门式办理”“一站式”综合服务平台建设，完善乡村有效治理体系。

　　（五）大力推进改革开放。高举新时代改革开放旗帜，弘扬改革创新精神，强化改革开放担当，敢为人先、先行先试，坚定不移将改革开放进行到底。

　　深化重点领域改革。深入推进市、县机构改革，确保在3月底基本完成。坚持以大卫生大健康理念统领医改工作，坚持“三医联动”改革方向，完善全民健康“四级共保”工程和医联体建设，把更多救命救急的好药纳入医保，提升慢性病一体化管理水平。加快全国集体林业综合改革试验区建设，深化集体林地“三权分置”和集体林权股权化、资本化运作等改革，继续推广林权按揭贷款、“福林贷”等林业金融产品。推动绿色金融改革，积极创新绿色金融产品，争创国家绿色金融综合改革创新试验区。巩固提升国家生态文明试验区建设成果，完善环境信用评价、环境责任保险以及排污权、碳排放权交易等体制机制，提升河湖长制管理水平。加快国资国企改革，注重从管企业向管资本转变，积极推进市属国有企业改革重组。深化基础教育“总校制”、教师“县管校聘”等办学体制机制改革，不断提升教育发展水平。

　　持续扩大对外开放。积极融入海上丝绸之路核心区建设，主动对接复制福建自贸区试验成果，完善“一带一路”投资合作项目库，引导海西重汽、海斯福化工、南方制药等一批优质企业“走出去”。培育进出口规模超亿元企业10家以上，力争生产型出口增长10%以上。用好中国国际进口博览会平台，提高进口质量和水平。进一步拓展对外交流交往，办好第十五届林博会，密切与港澳地区的沟通联系，促进明台合作，加强与国外友城友好往来，鼓励海外华侨华人及其社团来明投资创业。

　　（六）大力推进民生社会事业发展。坚持以人民为中心的发展思想，把人民群众对美好生活的向往作为奋斗目标，老百姓想什么，我们就干什么，不断增强人民群众的获得感、幸福感、安全感。

　　补齐社会事业短板。着力优化教育布局，缓解供求矛盾，实施公办幼儿园、城区中小学扩容等工程，新增学位1.16万个，逐步消除义务教育阶段“大班额”；推进小学“强基”、初中“壮腰”、高中“筑梦”基础教育质量创新工程，加强名师名校长培养，实施乡村教师支持计划，开展公费师范生培养，深化高等教育内涵式发展，支持三明学院硕士学位授予培育单位建设，强化职业教育产教融合校企合作，促进特殊教育、终身教育、老年教育加快发展。组织实施“健康三明2030”行动计划，加快产科、儿科、精神科等短板学科建设，强化重点特色专科建设，抓好疾病预防控制，传承发展中医药事业，推进市级全民健康信息化服务平台和县域医疗卫生信息化建设，全市医疗机构新增床位600张。加快国家居家和社区养老服务改革试点城市建设，推进农村养老服务改革，创建老年人健身康乐家园，新增各类养老床位1000张以上，力争农村各类养老服务设施覆盖率超55%。

　　织牢民生保障网络。把稳定就业摆在突出位置，重点解决好高校毕业生、农民工、退役军人等群体就业，继续实施“十百千”创业创新计划，新增城镇就业1.8万人以上。落实基本养老保险待遇等相关政策，加快推进全民参保计划，做好社会保险登记工作。健全社会救助体系，加强留守儿童保障和关心下一代工作，发展残疾人、社会福利和慈善事业。开展“治欠保支提升年”活动，强化拖欠工资问题源头治理。

　　推动文化共建共享。大力弘扬社会主义核心价值观，深入开展以“全国文明城市”和全国“志愿之城”为统揽的群众性精神文明创建活动，推进“诚信三明”建设，树立良好社会风尚。着力增加公共文化服务供给，加快县级融媒体中心建设，打造一批融合地方特色的红色文化、客家文化、朱子文化文学艺术精品，加强文化遗产保护工作，开展科技文化卫生“三下乡”集中示范服务活动，编好地方志、综合年鉴。支持开展“月月有比赛”和“泰宁环大金湖马拉松赛”等体育赛事，抓好全民健身体育场馆设施建设，重视老年人体育事业，办好第七届三明市运动会。

　　提升社会治理水平。深入推进扫黑除恶专项斗争和五个专项治理，加强信访问题化解息访，完善社会矛盾多元化解机制，提升“平安三明”建设成效。深化“七五”普法，推进“法律六进”和法治创建活动。创新食品药品监管方式，用“四个最严”确保人民群众“舌尖上的安全”。贯彻落实地方党政领导干部安全生产责任制实施细则，坚决防范遏制重特大生产安全事故发生。做好防汛防台风、地震、水文、气象、地质等工作，提升防灾减灾救灾能力水平。强化与各民主党派、工商联、无党派代表人士联系，提高民族、宗教、侨务工作水平，支持工会、共青团、妇联等群团组织发挥作用，抓好第四次全国经济普查。加强人防工作，完善拥军优属政策和国防动员机制，推动军民融合加快发展。

　　三、加快建设新时代人民满意政府

　　我们要按照全面从严治党要求全面从严治政，着力对标“五个过硬”，努力提升“八项本领”，大力倡导“务实高效创新廉洁”的工作作风，做到在政治立场上更加坚定清醒、在执行落实上更加有力有效、在为民服务上更加用心贴心。

　　（一）始终注重政治过硬。把政治建设摆在首位，增强政治觉悟、政治能力、政治担当，牢固树立“四个意识”，坚定“四个自信”，做到“两个维护”，严守政治纪律和政治规矩，始终在思想上政治上行动上同以习近平同志为核心的党中央保持高度一致；持续学深悟透习近平新时代中国特色社会主义思想，进一步打牢理想信念之基，始终做共产主义远大理想和中国特色社会主义共同理想的坚定信仰者和忠实实践者；确保政令畅通，坚决贯彻落实党中央国务院和省委省政府、市委决策部署，决不打任何折扣，决不耍任何小聪明，决不搞任何小动作，全力推动各项重大决策部署一抓到底、落地见效。

　　（二）始终注重担当作为。把担当负责、奋发有为作为履职尽责第一要务，强化改革创新，主动思变、积极应变，探索更多好机制、好做法、好经验，不断向改革创新要空间、要活力、要效益。强化狠抓落实，着力在务实戒虚上下功夫，坚持“能在现场就不在会场”“能在第一线就不在办公室”，把更多精力放在解决一个个实际问题上来，把更多时间花在推动一项项工作落实上来，以钉钉子精神把定下的事干实干好，把承诺的事办成办妥。强化担当负责，发扬斗争精神，鼓励创造性贯彻落实，完善正向激励、容错纠错机制，旗帜鲜明为敢于担当的干部担当，为敢于负责的干部负责，激发干部干事创业精气神。

　　（三）始终注重清正廉洁。把为民务实清廉作为政府第一形象，坚持依法行政，全力保障宪法和法律实施，主动接受人大法律监督和工作监督、政协民主监督，接受社会和舆论监督，健全依法决策机制，严格规范公正文明执法，提升政府立法能力，加强法治政府建设。转变政府职能，大幅减少政府对资源的直接配置，凡是市场能自主调节的就让市场来调节，企业能干的就让企业来干。加强作风建设，弘扬“马上就办、真抓实干”优良作风，集中整治形式主义、官僚主义，今年市级对基层的督查检查考核事项、频次同比均减少50%以上，把干部从一些无谓的事务中解脱出来。坚守廉洁底线，严格落实中央八项规定及实施细则精神和省、市实施办法，健全重点领域廉政风险防控机制，全面实施预算绩效管理，更好发挥审计监督作用，坚决整治侵害群众利益的不正之风和腐败问题。

　　各位代表，新时代是奋斗者的时代，幸福都是奋斗出来的，我们都是追梦人。让我们更加紧密地团结在以习近平同志为核心的党中央周围，高举中国特色社会主义伟大旗帜，在省委省政府和市委的坚强领导下，金戈铁马、奋力拼搏，抓铁有痕、踏石留印，为推进高质量发展落实赶超、决胜全面建成小康社会而努力奋斗！
